# Intratumor microbiome: selective colonization in the tumor microenvironment and a vital regulator of tumor biology

**DOI:** 10.1002/mco2.376

**Published:** 2023-09-26

**Authors:** Mingjie Jiang, Zhongyuan Yang, Juanjuan Dai, Tong Wu, Zan Jiao, Yongchao Yu, Kang Ning, Weichao Chen, Ankui Yang

**Affiliations:** ^1^ Department of Head and Neck Surgery Sun Yat‐Sen University Cancer, State Key Laboratory of Oncology in South China, Collaborative Innovation Center of Cancer Medicine Guangzhou P. R. China; ^2^ Department of Intensive Care Unit Sun Yat‐Sen University Cancer, State Key Laboratory of Oncology in South China, Collaborative Innovation Center of Cancer Medicine Guangzhou P. R. China

**Keywords:** colonization, microbiome, mutational landscape, mutual regulation, therapy response, tumor microenvironment

## Abstract

The polymorphic microbiome has been proposed as a new hallmark of cancer. Intratumor microbiome has been revealed to play vital roles in regulating tumor initiation and progression, but the regulatory mechanisms have not been fully uncovered. In this review, we illustrated that similar to other components in the tumor microenvironment, the reside and composition of intratumor microbiome are regulated by tumor cells and the surrounding microenvironment. The intratumor hypoxic, immune suppressive, and highly permeable microenvironment may select certain microbiomes, and tumor cells may directly interact with microbiome via molecular binding or secretions. Conversely, the intratumor microbiomes plays vital roles in regulating tumor initiation and progression via regulating the mutational landscape, the function of genes in tumor cells and modulating the tumor microenvironment, including immunity, inflammation, angiogenesis, stem cell niche, etc. Moreover, intratumor microbiome is regulated by anti‐cancer therapies and actively influences therapy response, which could be a therapeutic target or engineered to be a therapy weapon in the clinic. This review highlights the intratumor microbiome as a vital component in the tumor microenvironment, uncovers potential mutual regulatory mechanisms between the tumor microenvironment and intratumor microbiome, and points out the ongoing research directions and drawbacks of the research area, which should broaden our view of microbiome and enlighten further investigation directions.

## Graphical abstract

Interplay between intratumor microbiome and the tumor microenvironment. (A) Routes by which the microbiome enters the tumor microenvironment. Tumors that directly contact with the external microenvironment could be colonized by microbiome from the resident bacteria. Microbiome could also be transmitted from the blood stream or other physical channels. (B) The tumor microenvironment is hospitable for the colonization of microbiome. The characteristics of the tumor microenvironment select suitable bacteria to reside and the interaction of microbiome with the tumor microenvironment promotes microbiome colonization. (C) Intratumor microbiome promotes cancer initiation and progression. The intratumor microbiome promotes cancer development via inducing genetic and signaling changes and modulating the tumor microenvironment through various mechanisms as presented. (D) Mutual regulation of the intratumor microbiome and the adopted therapy. The cancer therapy strategies change the composition of intratumor microbiome, while microbiome also actively influences the efficacy of cancer therapy.

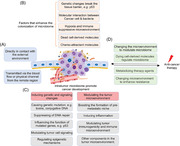

## 1 INTRODUCTION

Microorganisms are the important component of our body, with the cumulative microbial genome exceeding the human genome by a factor of more than 100.^1^ With the help of next‐generation sequencing (NGS), we are surprised to find that the microbiome also resides in the tumor tissue, even in those that do not directly connect with the external microenvironment.^2^ Moreover, the microbiome would change along tumor initiation,^3^ and bacteria in the primary tumor could be found in the metastatic tissues or even in the patient‐derived xenograft (PDX) tissues after several successive passages.^4^ These results indicate the supportive force in the tumor microenvironment for the microbiome. The unique microenvironment within tumors may select certain species to reside.

The host microbiome is vital in regulating physiological and pathological processes.^5^, ^6^, ^7^, ^8^ Of note, the role of microbiome in regulating the biology of cancer has recently been underscored,^9^ and the polymorphic microbiome has been proposed as a new hallmark of cancer.^10^ Many researches have revealed the direct interaction between microbiome and tumor cells.^11^ As a newly found player in the tumor microenvironment, numerous studies have proven that the composition of tumor microbiota is significantly different from that of adjacent normal tissues^12^ and is widely associated with clinical features of patients (Table 1).^13^, ^14^, ^15^, ^16^, ^17^, ^18^, ^19^, ^20^, ^21^, ^22^, ^23^, ^24^, ^25^, ^26^, ^27^, ^28^, ^29^, ^30^, ^31^, ^32^, ^33^ These results highlight the potential role of intratumor microbiome in regulating cancer development. Thus, the roles and mechanisms of mutual regulation among intratumor microbiome, tumor cells, and other components of the tumor environment urgently need to be uncovered. Moreover, the currently adopted anti‐cancer therapies have been revealed to change the composition of intratumor microbiome, while intratumor microbiome could influence therapeutic effects. Thus, targeting or exploiting the intratumor microbiome has become the new research direction for better tumor control.

**TABLE 1:** Examples of the clinical significance of intratumor microbiome.

Tumor type	Microbiome features or bacteria species	Clinical significance	Reference
Neuroblastoma	Abundance of intratumor microbiome	High‐risk patients; The index is better than the conventional Children's Oncology Group (COG) risk group assignment	^13^
Nasopharyngeal carcinoma	Load of intratumor microbiome	Poor prognosis; Negatively associated with T‐lymphocytes infiltration	^14^
Head and neck squamous cell carcinoma	*Fusobacterium nucleatum*	Less frequent lymph node invasion, and a trend for a lower recurrence rate and longer overall survival; Associated with older age, less alcohol and combined alcohol plus tobacco consumption	^15^
	Microbiome signature	Host‐gender, age, tumor stage, and histologic grade	^16^
Oral squamous cell carcinoma	Periodontitis‐correlated taxa, including *Fusobacterium*, *Dialister*, *Peptostreptococcus*, *Filifactor*, *Peptococcus*, *Catonella*, and *Parvimonas*	Good diagnostic power	^17^
	Phylum *Firmicutes*, phylum *Actinobacteria*, class *Fusobacteriia*, order *Fusobacteriales*, family *Fusobacteriaceae*, and genus *Fusobacterium*	Clinical survival time and patient status	^18^
Thyroid cancer	Enriched tumor bacterial diversity	Higher invasive tumors; Specific bacterial genus was correlated with the level of thyroid hormones and auto‐immune antibodies	^19^
Esophageal carcinoma	*F. nucleatum*	High abundance with poor therapy response and patient survival	^20^
		High abundance with poor Relapse‐free Survival (RFS) and displayed poor chemotherapeutic response	^21^
	*Firmicutes*, *Proteobacteria*	Tumor subtype, stage, and survival status	^22^
Lung cancer	*Haemophilus parainfluenzae*, *Serratia marcescens*, *Acinetobacter jungii*, *Streptococcus constellation*	First‐line treatment efficacy and survival; Predict 2‐year survival with an accuracy rate of 90.7%	^23^
	Bacterial burden	Immune cell abundance, histology type, patient medical history, mutation profile, the expression level of β‐catenin	^24^
Pancreatic cancer	Alpha‐diversity; *Pseudoxanthomonas*, *Streptomyces*, *Saccharopolyspora*, *Bacillus clausii*	Predict cancer survival	^25^
	*Megasphaera*	Survival time; Associated with the metabolic pathways in the tumor microenvironment and efficacy of immune therapy	^26^
Gastrointestinal cancer, particularly colorectal cancer	*Alistipes*, *Blautia*, *Pasteurellales*, and *Porphyromonas*	Clinical characteristics of patients	^27^
Colorectal cancer	*Fusobacterium nucleated*	Worse prognosis	^28^
Cholangiocarcinoma	*Gammaproteobacteria*	Gemcitabine‐ and cisplatin‐resistant; Different metabolome	^29^
Prostate cancer	*Listeria monocytogenes*, *Methylobacterium radiotolerans*, *Xanthomonas albilineans*, *Bradyrhizobium japonicum*	Negatively correlated with Gleason score, Tumor‐nodes‐metastasis (TNM) stage, Prostate‐specific Antigen (PSA) level, and Androgen Receptor (AR) expression	^30^
Non‐muscle‐invasive bladder cancer	*Pseudomonas*, *Staphylococcus*, *Corynebacterium*, *Acinetobacter genera*	Recurrence	^31^
Cutaneous melanoma	*Lachnoclostridium genus*	Overall survival; CD8^+^ T cells, CXCL9, CXCL10, and CCL5 expression	^32^
Squamous cell vulvar carcinoma	*F. nucleatum*, *Pseudomonas aeruginosa*	Poor prognosis and highly infiltrating of neutrophils	^33^

In this review, by comprehensively analyzing research results concerning intratumor microbiome, we presented the interplay between tumor cells and intratumor microbiome, mainly the bacteriome. We presented that as a component of the tumor microenvironment, the composition of intratumor microbiome is actively shaped by tumor cells and adopted anti‐cancer therapies. The tumor microenvironment is hospitable for the colonization of certain microbiomes. And conversely, the intratumor microbiome contributes to genetic, epigenetic, and signaling changes in tumor cells, modulates the tumor microenvironment, and influences the efficacy of therapies. These facts highlight the vital role of intratumor microbiome in tumor initiation, progression, and therapy resistance, which could be a therapeutic target or exploited as a weapon to fight against cancer. Finally, we presented the ongoing research highlights and discussed the drawbacks in the research field of intratumor microbiome, in order to finally put forward the potential investigation directions in the future. In summary, this review emphasized that the interplay between intratumor microbiome and the tumor microenvironment, uncovered and summarized the underlying mechanisms, and presented the ongoing research directions and shortcomings in the research area, which could innovate more meaningful investigations.

## 2 THE COLONIZATION OF MICROBIOME IN THE TUMOR MICROENVIRONMENT

**FIGURE 1:**
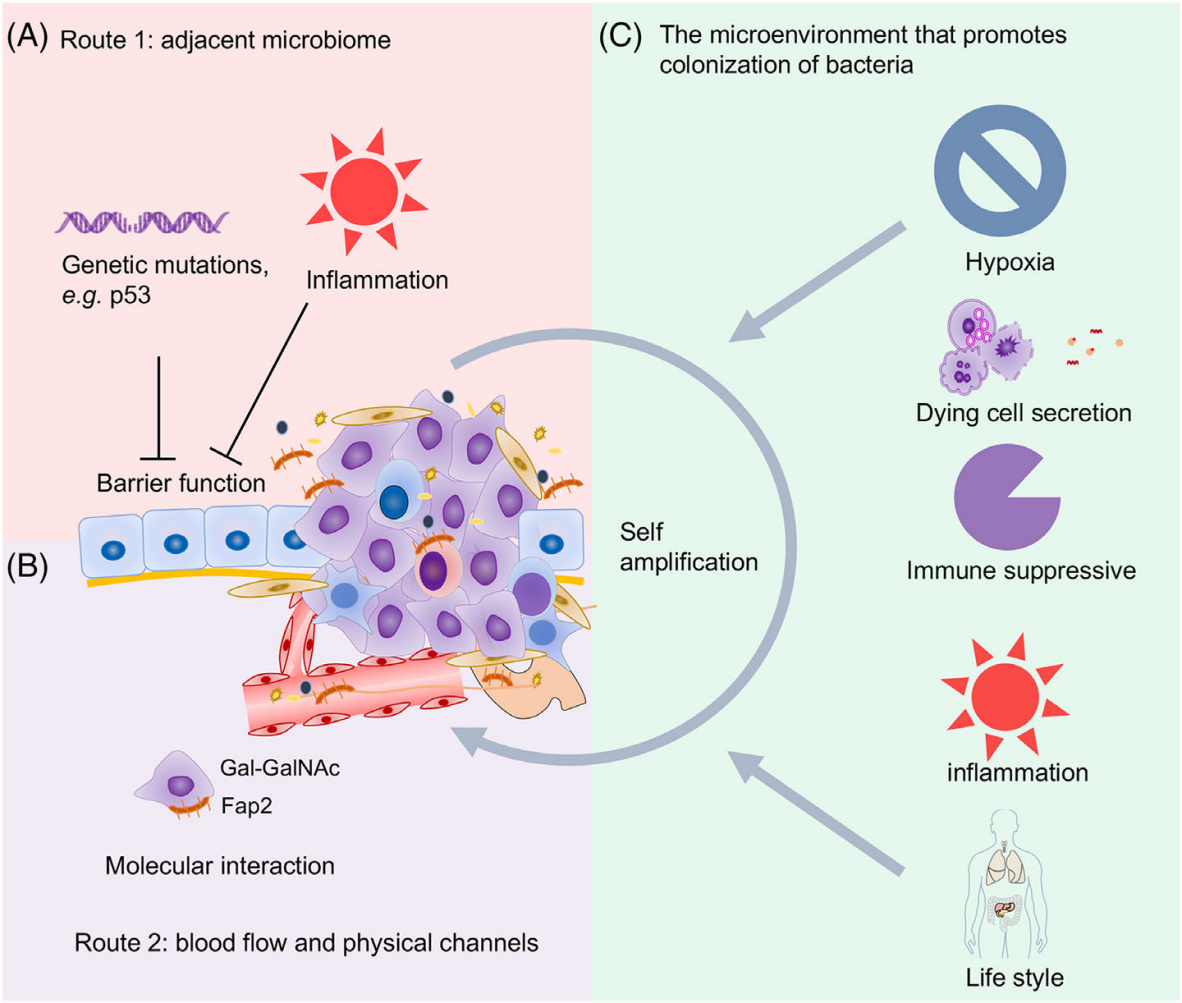
Mechanisms of tumor microenvironment in shaping the composition of intratumor microbiome. The intratumor microbiome could enter the tumor tissue via several routes. The adjacent tissue‐resident microbiome could colonize the tumor tissue via the disrupted epithelial barrier induced by tumor‐associated genetic mutations or inflammation (A). Moreover, the microbiome may colonize the tissue from the remote region via blood flow or physical channels. And the microbiome may select to specifically reside in the tumor tissue via molecular interactions (B). More importantly, the characteristics of the tumor microenvironment are hospitable for certain microbiome (C), resulting in the colonization of microbiome in tumor tissues as well as the organ‐ and tumor type‐specific composition of intratumor microbiome.

Innovated by the finding that Rous sarcoma virus caused sarcoma development in chicken,^34^ oncologists have once spared no effort to search for onco‐microorganisms. Although several microorganisms were revealed to be associated with cancer,^35^ most cancers failed to be proven to be associated with microorganisms at that time, mainly due to the low biomass of microorganisms within cancer tissues. Many tissues from which the tumor arose were traditionally considered to be sterile. Thus, the existence of intratumor microbiome has been neglected and unrecognized for a long time. Luckily, with the advance of technologies, mainly NGS, it has been revealed that most tumor tissues contain microbiomes.^2^ Numerous studies have revealed differences in the composition of microbiome between healthy tissues and tumors (Table 1). Nevertheless, how and why the microbiome colonizes the tumor are not fully illustrated. In this review, we first systematically discussed the routes and mechanisms for the colonization of microbiome in tumor tissues (Figure 1).

### 2.1 Routes of the colonization of intratumor microbiome

Generally, there are several routes by which bacteria may colonize the tumor. First, tumors arise from the organs that directly connect with the external environment and may have bacteria from the resident microbiome; for example, nasopharyngeal microbiota was the main origin of intratumor bacteria in nasopharyngeal cancer.^14^ Especially, the tumor may disrupt the barrier of epithelium or mucosa, which could promote the colonization of resident microbiome. Tumors harboring *TP53* mutations, which can impair epithelial function, have a unique bacterial consortium, mainly the *Acidovorax temporans* in lung cancer.^36^ A recent study revealed that the biopsy site, rather than primary tumor type, was associated with microbiome diversity, highlighting the role of adjacent environment.^37^ Second, bacteria may be transmitted from remote regions of our body to colonize tumor tissue via blood stream or other physical channels. Bacteria could be transmitted from the gut to pancreas as evidenced by Green Fluorescent Protein (GFP)‐labeled *Escherichia coli* and Carboxyfluorescein Succinimidyl Ester (CFSE)‐labeled *Enterococcus faecalis*, which were orally administered and detected in the pancreas.^38^ Same bacteria could be transmitted to tumor tissues via different mechanisms. For example, in colorectal cancer (CRC), both routes significantly contribute to the composition of intratumor microbiome. Barrier deterioration induced by CRC‐initiating genetic lesions or inflammation results in the invasion of microbiota, which contributes to tumor development.^39^ Oral‐derived microbiome, including four *Fusobacterium* spp., was found to be enriched in colorectal tumors^40^, ^41^; although the reason why these bacteria translocated from the oral to gut was not fully illustrated, several studies have revealed the potential way. A previous study revealed that the common oral bacterium *Fusobacterium nucleatum* was more likely to translocate to CRC using the hematogenous route.^42^ Another study also revealed that oral gavage of *F. nucleatum* was sufficient to promote colorectal tumor development, suggesting that transmission via the gastrointestinal tract was also possible.^43^

### 2.2 Hospitable tumor microenvironment for the colonization of intratumor microbiome

Despite the route by which the microbiome resides in tumor tissues, the selective colonization of certain bacteria may be ascribed to tumor tissues are permissive places for bacteria to reside and proliferate. When the bacteria *Bifidobacterium breve* was administered through tail vein injection or oral gavage, it could be specifically detected later in the subcutaneous tumor tissues.^44^ Using high‐resolution in vivo bioluminescent imaging, after intravenous administration of bacteria including non‐pathogenic commensal bacteria *E. coli* and *B. breve*, or *Salmonella typhimurium*, these bacteria were detected specifically in subcutaneous tumors of mice.^45^ These facts hint that the tumor tissue or certain microenvironmental factor could recruit specific species. Of note, the fact that *F. nucleatum* and its related bacteria were also detected in the metastatic CRC and PDX tissues after several successive passages indicated that the tumor microenvironment might be a “greenhouse” for certain bacteria.^4^ More specifically, certain bacteria could colonize the tissue through the interaction of molecules between bacteria and tumor tissues. *F. nucleatum* colonized breast cancers via the Fap2‐dependent binding to tumor‐displayed Gal‐GalNAc.^46^ Intravenously administrated *F. nucleatum* localized to mouse tumor tissues in a Fap2‐dependent manner.^47^ However, most of the results displayed are found in *F. nucleatum* due to the intensive investigation of this bacteria. More results regarding the molecular interaction between intratumor microbiome and tumor cells will be found in the future as the research going forward.

Conversely, the bacteria could even help create a suitable microenvironment for themselves to reside in the tumor tissues. After systematic administration of *S* *almonella enterica* **serovar Typhimurium**, tumor necrosis factor‐alpha was rapidly elevated in the blood, which significantly promoted bacteria to be flushed into the tumor followed by tumor necrosis formation, bacterial growth, and infiltration of neutrophilic granulocytes.^48^

### 2.3 Factors that cause the similarities and differences in the intratumor microbiome

Overall, based on the biology of cancer and the characteristics of bacteria, the colonization of bacteria in the tumor may further ascribed to the following reasons. First, the tumor microenvironment is usually hypoxic,^49^ which may potentiate the survival and proliferation of anaerobic and facultative anaerobic bacteria. In addition, different tissues may have different oxygen supply levels, which may account for the differences in the residing bacteria. Most cancers may select anaerobic bacteria but lung tumors could favor aerobic bacteria. Second, tumor tissues often have necrotic regions, and dying tumor cell‐derived components have been revealed to modulate the composition of microbiome,^50^ the reason for which may be that the necrotic region could provide nutrients and other molecules for the bacteria to outgrowth. Third, tumor cells may release chemo‐attractant compounds, which could recruit bacteria. Last, the tumor microenvironment is usually immune suppressive, which may protect the bacteria against the clearance by immune system.^51^ Different immune subtypes may thus select different bacteria.^52^, ^53^, ^54^ In colon cancer models, depletion of neutrophils led to increased number of bacteria in tumors and proliferation of tumor cells accompanied by increased tumor cell DNA damage and an inflammatory response mediated by interleukin‐17 (IL‐17),^55^ indicating that the intratumor microbiome is under the active surveillance of immune system and that the immune suppressive microenvironment may contribute to the colonization of bacteria. Conversely, neutrophilic inflammation seems to be permissive for tumor‐promoting bacteria growth in squamous cell vulvar carcinoma,^33^ indicating the context‐dependent role of immune cells. As evidence, using in situ spatial‐profiling technologies and single‐cell RNA sequencing, it was revealed that bacterial communities populated microniches that were less vascularized and highly immuno‑suppressive in oral squamous cell carcinoma and CRC.^56^ When comparing different tissue parts of oral cancers, the microbiome of tumor tissues had a greater alpha diversity, while minor differences in diversity existed between outer tumor tissues and inner tumor tissues. Nevertheless, *Fusobacterium*, *Neisseria*, *Porphyromonas*, and *Alloprevotella* were more abundant in outer tumor tissues, while *Prevotella*, *Selenomonas*, and *Parvimonas* were enriched in inner tumor tissues.^57^

### 2.4 Risk factors for cancer influence the colonization of intratumor microbiome

Furthermore, the microbiome could also be influenced by factors that are associated with the characteristics of patients, especially the known etiologies of cancers, indicating the important role of intratumor microbiome in potentiating tumor initiation. Intratumor microbiome in lung cancer was associated with age, gender, and other clinical features.^58^ And the abundance of *A. temporans* was also higher in smoking‐associated lung cancers, indicating that smoking helps to create an environment for the growth of these bacteria and these bacteria may contribute to regulating the tumor biology.^36^ Patients with pancreatic cancer who were males and smokers showed the presence of potentially cancer‐promoting or immune‐inhibiting microbes in the pancreas, and had poorer prognosis.^59^ The etiologies of liver cancer, including alcohol and hepatitis B, significantly influenced the composition of the liver microbiome, which further participated in the tumorigenic process.^60^ Together, these data all support that the intratumor microbiome is influenced by many factors, and they could participate in the process of cancer progression.

## 3 MECHANISMS OF INTRATUMOR MICROBIOME IN MODULATING TUMOR INITIATION AND PROGRESSION

**FIGURE 2:**
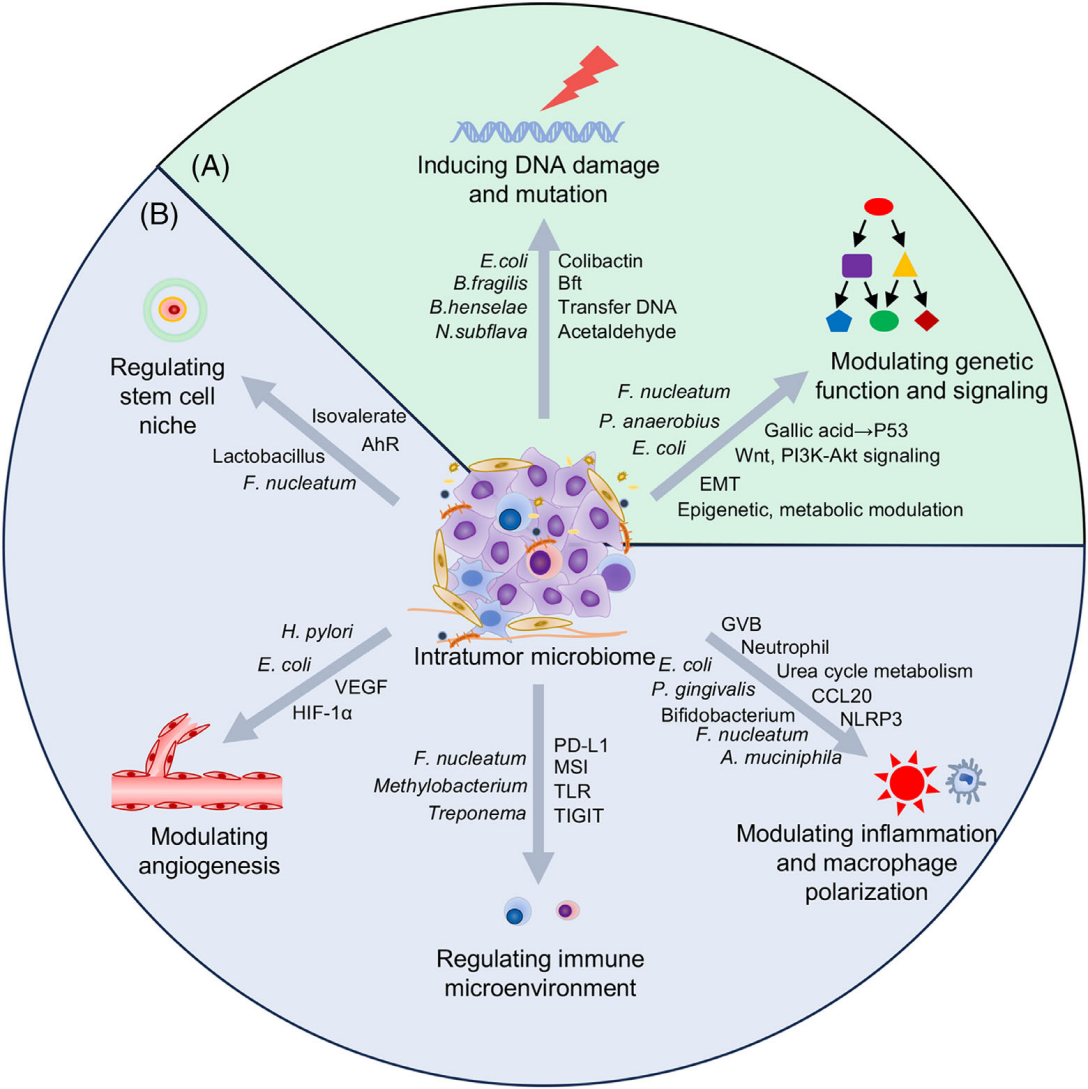
Mechanisms of intratumor microbiome in regulating the tumor microbiology. The intratumor microbiome could regulate the tumor biology via various mechanisms, including directly regulating cancer cells (A) and modulating the tumor microenvironment (B). The prototype bacteria that were found to modulate tumor biology are listed on the left of the arrow, while the revealed mechanisms or molecules mediating the effects are listed on the right.

Numerous evidences have shown that the intratumor microbiome is associated with clinical features and prognosis of patients (Table 1). In the tumor microenvironment, the amount of microbiome was usually elevated, but the diversity of which was downregulated, with some bacteria species dominating the microenvironment. The effects of intratumor microbiome in promoting cancer development have been thoroughly discussed elsewhere,^61^, ^62^ and we herein will focus on the underlying mechanisms (Figure 2).

### 3.1 Intratumor microbiome modulates genetic changes and signaling in tumor cells

#### 3.1.1 Intratumor microbiome induces genetic mutations and is associated with tumor genetics

Genetic mutation is considered as the driving force of cancer development.^63^, ^64^ Numerous researches have revealed that the microbiome directly induced DNA damage and thus accelerated tumor initiation. As a high‐risk population for developing CRC, patients with familial adenomatous polyposis presented patchy bacterial biofilms composed predominantly of *E. coli* and *Bacteroides fragilis*, which encoded oncotoxins colibactin (clbB) and Bacteroides fragilis toxin (bft).^65^ Colibactin alkylates DNA with an unusual electrophilic cyclopropane.^66^ The bacteria in the bacterial biofilms induced IL‐17 expression and DNA damage in colonic epithelium, which further led to faster tumor onset and increased mortality of patients.^65^ Distinct mutational signatures were found before and after the exposure to pks^+^ *E. coli* in colon organoids, and the signature after exposure was detected in patients from two independent cohorts, indicating that the underlying mutational process results directly from the previous exposure to pks^+^ *E. coli*.^67^ However, findings also revealed that the majority of CRC cases did not have the mutational signature, and many patients who have pks^+^ *E. coli* did not develop CRC. This is in consistence with the fact that the tumor development is a complex process influenced by many factors, and that the genetic mutation alone is not sufficient.^68^ Similarly, *E. coli* carrying the pks‐island‐produced colibactin also induced a novel mutational signature in oral squamous cell carcinoma and in other mucosal tumor types.^69^ Furthermore, in oral and even upper aerodigestive tract carcinomas, the residing of certain bacteria, including *Neisseria subflava*, *Streptococcus mitis*, *Candida albicans*, *Glabrata*, etc., that are generally non‐pathogenic, could convert alcohol into carcinogen acetaldehyde and thus play a subtle role in alcohol‐related carcinogenesis in human oral cancer.^70^, ^71^ Acetaldehyde causes DNA damage and blocks DNA repair, and is usually further metabolized by aldehyde dehydrogenase (ALDH) in the liver, while in the oral cavity, the activity of ALDH is relatively low, resulting in the long existence of acetaldehyde and more deteriorating results.^72^, ^73^ Conversely, alcohol ingestion resulted in increased levels of *Neisseria*.^74^ And the fact that *Neisseria* is more abundant in outer tumor tissues^57^ is also consistent with the role of *Neisseria* in promoting carcinogenesis. Moreover, pathogenic bacteria such as *Bartonella henselae* could transfer their conjugative DNA into nucleus of human cells by the VirB/VirD4 type IV secretion system,^75^ which could be another source of genetic mutations. Taken together, these results reveal that the bacteria actively participate in modulating genetic changes and promoting cancer initiation via different mechanisms in different organs.

In addition, there are large amounts of evidences indicating that the composition of microbiome is associated with the genetic background of the tumor. By analyzing the mutational signature and intratumor microbiota in CRC, Okuda et al. found that *Fusobacterium* was associated with many mutated genes, as well as genes in cell cycle‐related pathways.^76^ A high abundance of *Campylobacter* was associated with the mutational signature, including failure of double‐strand DNA break repair.^76^ In biopsies with or without *KRAS* mutation or microsatellite instability (MSI), there was a significant difference in intratumor microbiota composition,^77^ highlighting the association between intratumor microbial heterogeneity with genetic alteration. Moreover, significantly less microbial diversity was found in gastric cancer tissues compared with non‐malignant tissues in both MSKCC and TCGA cohorts, and MSI‐high gastric cancer had distinct microbial enrichment compared to other molecular subtypes.^78^ CRC‐associated intratumor genera, including *Dialister* and *Casatella*, were associated with the MSI status,^79^ indicating the higher mutation rate associated with these bacteria. These facts further support the role of intratumor microbiome in regulating the tumor mutational landscape.

#### 3.1.2 Intratumor microbiome modulates functions of genes

Besides directly accelerating genetic mutations, the intratumor microbiome could greatly influence the function of mutated genes, or even change their function, in tumor cells. The effects of mouse *p53* hotspot mutations (R172H and R270H, corresponding to R175H and R273H in humans, respectively) on CRC models were controlled by the microbiota. In environments that lacked microbiota, *p53* hotspot mutation exerted tumor suppressive effect, while in microenvironments presented with microbiota, the mutant *p53* conversely exerted oncogenic properties.^80^ Mechanistical investigations revealed that this controversial effect was mediated by the metabolite derived from the microbiome—gallic acid, which reinstated the Transcription Factor‐4 (TCF4)–chromatin interaction and the hyperactivation of Wnt signaling.^80^ Although similar researches concerning the role of microbiome in regulating gene functions are still lacking, this work strongly indicates that the role of intratumor microbiome should be noticed and worth further investigation.

#### 3.1.3 Intratumor microbiome regulates cellular signaling

Intratumor microbiome could further modulate tumor cell signaling to potentiate cancer development. *F. nucleatum* was revealed to directly bind to E‐cadherin, activate β‐catenin signaling, and differentially regulate the inflammatory and oncogenic responses in CRC.^28^ And *Peptostreptococcus anaerobius* surface protein PCWBR2 directly interacted with colonic cancer cell lines via α_2_/β_1_ integrin, which induced the activation of the PI3K–Akt pathway in CRC cells and led to increased cell proliferation and nuclear factor‐kappa B (NF‐κB) activation.^81^ Vergara et al. further illustrated that microbiota‐induced epithelial–mesenchymal transition (EMT) and inflammation were the common molecular mechanisms that induced tumor initiation and progression.^82^ In addition, in the muscle invasive bladder carcinoma, a variety of microbes, including *E. coli*, butyrate‐producing bacterium SM4/1, and a species of *Oscillatoria*, were associated with the expression of classical EMT‐associated genes, including E‐cadherin, vimentin, SNAI2, SNAI3, and TWIST1.^83^ And airway microbiota enriched in lung cancer patients, mainly the *Streptococcus* and *Veillonella*, contributed to the upregulation of the PI3K pathway in lung cancer.^84^ Interestingly, intratumor microbiome accompanied cancer cells during the metastasis process in breast cancer, and could promote cancer cell metastasis by enhancing cell viability through reorganization of the actin cytoskeleton.^85^ Intervention of tumor microbiota using antibiotics impeded tumor metastasis, but did not inhibit the growth of primary tumors.^85^ Besides, in oral cancer, the *F. nucleatum*‐secreted outer membrane vesicles enhanced migration and metastasis of cancer cells via activating intracellular autophagy pathways.^86^ *F. nucleatum* infection in the pancreas induced the expression of cytokines, including Granulocyte‐macrophage Colony‐stimulating Factoe (GM‐CSF), etc., from normal and cancerous pancreatic cells to promote the aggressive phenotype of pancreatic cancer cells.^87^ Together, these data reveal that the microbiome could regulate the signaling of cancer cells, which could be another mechanism that supports cancer development.

#### 3.1.4 Intratumor microbiome influences the epigenetic status of cancer cells

Moreover, there is also much evidence showing that microbiome regulates the biology of cancer cells via epigenetic mechanisms. In stomach cancer, the intratumor microbiome, especially *Kytococcus sedentarius* and *Actinomyces orisinteracted*, was strongly associated with methylation changes in immune‐related genes of cancer cells and regulated gene expression.^88^ In esophageal cancer, the presence of *F. nucleatum* was associated with LINE‐1 hypomethylation, which was associated with poor prognosis.^89^ Butyrate, a common metabolite of the gut microbiota, exerted antitumor effect via accumulating in the CRC cells due to the Warburg effect (due to the Warburg effect, butyrate is not consumed within the cancer cells) and thus functions as a histone deacetylase inhibitor to stimulate histone acetylation and affect apoptosis and cell proliferation.^90^

#### 3.1.5 Intratumor microbiome changes the metabolism of cancer cells

The intratumor microbiome can also modulate the tumor biology via influencing metabolism. In intrahepatic cholangiocarcinoma, the intratumor microbiota *Paraburkholderia fungorum* was significantly enriched in paracancerous tissues, which could inhibit tumor growth through alanine, aspartate, and glutamate metabolism.^91^ And in breast cancer, integrating bulk and single‐cell RNA sequencing data revealed that the intratumor microbiome was significantly associated with metabolic heterogeneity in breast cancers, highlighting the need for further mechanistic investigations.^92^ These results indicate that the intratumor microbiome may influence many aspects of cancer cells, but may differ between different cancer types; more specific and comparative investigations are thus needed.

### 3.2 Intratumor microbiome regulates the tumor microenvironment to potentiate tumor development

It has been widely recognized that tissue microenvironment plays vital roles in cancer initiation and progression.^68^ Even though high frequency of tumor‐associated somatic mutations occurs in normal tissues, the tissues may not show any malignant phenotype.^93^, ^94^, ^95^, ^96^ Without suitable selective forces, mutated neoplasm cells remain dormant and could persist in the organ indefinitely without carcinogenesis.^97^ The microbiome in the tumor microenvironment also plays vital roles in inducing cancer carcinogenesis or metastatic cell outgrowth.^3^, ^98^ Only when stroma is exposed to the carcinogen could they lead to the carcinogenesis of mammary tumors, regardless of whether the epithelial cells are exposed to the carcinogen or not.^99^ The tumor environment is one of the determining factors in inducing cancer initiation and progression, and numerous researches have revealed the role of intratumor microbiome in regulating the tumor microenvironment (Figure 2).

#### 3.2.1 Intratumor microbiome enhances inflammation to promote tumor progression

Inflammation is one of the driving forces of carcinogenesis and metastatic colonization in various tissues,^100^, ^101^ and the microbiome significantly contributes to the establishment of an inflammatory microenvironment. In tet2‐deficient mice, a model of spontaneous hematopoietic malignancies, the development of pre‐leukemic myeloproliferation was highly dependent on microbial‐induced inflammation.^102^ Microbiota‐induced inflammation changed the epigenetic status of host cells, which further accelerated the process of inflammatory bowel disease and colitis‐associated CRC.^103^ Moreover, tumor‐resident bacteria *E. coli* further disrupted the gut vascular barrier (GVB) depending on the virulence regulator VirF. Then, bacteria disseminated to the liver, boosted the formation of a premetastatic niche, and favored the recruitment of metastatic cells.^104^ This phenomenon was validated in a clinical cohort. Patients with increased levels of PV‐1, a marker of impaired GVB, were associated with liver bacteria dissemination and metachronous distant metastases.^104^ These facts reveal the important role of microbiome‐related inflammation in cancer initiation and progression.

#### 3.2.2 Intratumor microbiome regulates the tumor immune microenvironment

The cancer immune microenvironment is the most well‐studied area of the interaction between intratumor microbiome and the tumor microenvironment. The ovarian cancer could be divided into immune‐enriched and immune‐deficient subtypes, which are correlated with the intratumor microbiome. And intratumor microbiome is closely associated with the prognosis of patients.^105^ The immune histopathological parameters such as PD‐L1 expression and tumor‐infiltrating lymphocytes were significantly associated with alpha and beta diversity of intratumor microbiome.^37^ Interestingly, the bacteria‐specific peptides were presented by tumor cells in both Human Leukocyte Antigen (HLA)‐I and HLA‐II molecules. T cells that specifically recognize these bacterial antigens were identified in the tumor microenvironment. Recurrent bacterial peptides in tumors from different patients, as well as in different tumors from the same patient, were identified.^106^ These findings suggest that bacteria actively participate in modulating the tumor immunogenicity and tumor microenvironment and highlight a therapeutic direction.

Furthermore, the intratumor microbiome is highly associated with the immune landscape of the tumor tissues. Multi‐omics analysis proved that intratumor microbiome was highly associated with tumor immune landscape and respones to immune therapy.^107^ In gastric cancer, elevated intratumor *Methylobacterium* was significantly associated with poor prognoses and reduced CD8^+^ tissue‐resident memory T cells.^108^ In cutaneous melanoma, certain intratumor bacteria were associated with the infiltration of CD8^+^ cells and chemokines, including CXCL9, CXCL10, and CCL5, which were further correlated with overall survival (OS) of the patients.^32^ Microbial dysbiosis was also revealed to contribute to colon tumor susceptibility by hyperstimulating CD8^+^ T cells to promote chronic inflammation and early T‐cell exhaustion, which further reduced antitumor immunity.^109^ As the rising star of immune checkpoint molecules, PD‐L1 expression contributes to tumor immunosuppression and is a target for immune checkpoint blockade therapy. *F. nucleatum* in the CRC could enhance PD‐L1 expression by activating the STING signaling, which enhanced the antitumor effects of PD‐L1 blockade.^110^ And consistently, *F. nucleatum* in the CRC was also associated with T‐cell infiltration and the MSI status, which was further differentially related to immune response.^111^, ^112^ Nevertheless, most of these findings reveal the correlation but not the cause–effect mechanisms. Based on limited evidence that microbiome actively shapes the tumor microenvironment, we tend to believe that the intratumor microbiome is not just a bystander, and further research is highly needed.

Of note, the microbiome has been revealed to influence many types of immune and inflammatory cells in the microenvironment. The commensal microbiota promoted lung cancer development via stimulating Myd88‐dependent IL‐1β and IL‐23 production from myeloid cells, inducing the proliferation and activation of γδ T cells that produced IL‐17 and other effector molecules to promote inflammation and tumor cell proliferation.^3^ The abundant bacteria in pancreatic cancer tissues were also revealed to generate a tolerogenic immune program by differentially activating Toll‐like receptors in monocytic cells^38^; ablation of these bacteria attenuated the oncogenesis and progression of pancreatic cancer and enabled the efficacy of checkpoint‐targeted immunotherapy.^38^ Of note, *F. nucleatum* in the CRC tissues increased tumor multiplicity and selectively recruited tumor‐infiltrating myeloid cells, which generated a proinflammatory microenvironment that can promote tumor progression.^43^ And periodontal pathogen *Porphyromonas gingivalis* could promote colorectal tumorigenesis by recruiting myeloid cells and creating a proinflammatory tumor microenvironment.^113^ Similarly, in oral squamous cell carcinoma and CRC, single‐cell in situ spatial‐profiling technologies and single‐cell RNA sequencing showed that intratumor bacteria, including *Fusobacterium* and *Treponema*, enhanced cell heterogeneity and promoted cell migration and metastasis by changing cellular signaling and recruiting myeloid cells to bacterial regions.^56^ More specifically, *F. nucleatum* Fap2 could bind to human TIGIT and inhibit immune activity of natural killer (NK) and T cells.^114^ In addition, the colonization of *F. nucleatum* in CRC suppressed the accumulation of tumor infiltrating T cells and promoted tumor growth and metastatic progression, which could be suppressed by antibiotic treatment.^46^ And elevated *P. gingivalis* promoted pancreatic cancer initiation and progression by creating inflammatory microenvironment through recruiting neutrophils, which promoted cancer progression through releasing neutrophil elastase.^115^ Together, these results strongly support that intratumor microbiome actively regulates the tumor immune microenvironment to further contribute to cancer initiation and progression.

#### 3.2.3 Intratumor microbiome modulates status of macrophages

As an important component of tumor microenvironment and a vital regulator of tumor biology,^116^ macrophages are also regulated by the intratumor microbiome. In pituitary neuroendocrine tumors, intratumor bacteria were associated with more abundant microglia and the macrophages in nerve tissues, which further showed longitudinally branched morphology,^117^ indicating the regulatory role of intratumor microbiome on macrophages. Similarly, more abundantly infiltrated M2 macrophages were detected in bacteria‐dominant subtype in HBV‐related hepatocellular carcinoma, which further showed multiple upregulated metabolism pathways.^118^ Specifically, absence of beneficial bacteria with ureolytic capacity, such as *Bifidobacterium*, was accompanied by elevated urea cycle metabolism in colorectal tumor tissue; the urea then enter into macrophages and further skew macrophages toward a pro‐tumoral phenotype characterized by the accumulation of polyamines.^119^ Gut microbiome imbalance induced by dominant colonization of *E. coli* promoted the expression of CTSK, which then induced M2 macrophage polarization and CRC progression.^120^ Similarly, *F. nucleatum* in CRC promoted CCL20 expression in tumor cells, which further induced M2 macrophage polarization and enhanced the metastasis of cancer cells.^121^ Remotely, the indole‐producing bacteria in the gut microbiome‐derived indole compounds activated the AhR receptor in tumor‐associated macrophages to suppress tumor growth and intra‐tumoral infiltration of IFNγ^+^CD8^+^ T cells in pancreatic cancer.^122^ On the other hand, supplementation with *Akkermansia muciniphila*, a bacteria that was significantly reduced in CRC, suppressed colonic tumorigenesis and tumor progression by inducing the enrichment of M1‐like macrophages in an NLRP3‐dependent manner.^123^ These facts reveal the regulatory role of intratumor microbiome in regulating the infiltration and differentiation of the macrophages, which further contribute to regulating tumor biology.

#### 3.2.4 Intratumor microbiome influences angiogenesis

Besides, microbiome also participates in modulating the angiogenesis process.^124^ Dysregulated angiogenesis is a hallmark of cancer and contributes to tumor progression.^125^ Targeting the abnormal angiogenesis signaling has shown some therapeutic effects in cancer.^126^ During the development of normal tissue, microbes colonizing the mucosal surface are assigned the responsibility for regulating elaboration of the underlying microvasculature by signaling through bacteria‐sensing epithelial cells.^127^ As a vital onco‐bacteria, *Helicobacter pylori* was revealed to be associated with increased Vascular Endothelial Growth Factor (VEGF) expression and neo‐angiogenesis, which may contribute to *H. pylori*‐related gastric carcinogenesis.^128^ Quorum sensing peptides produced by diverse commensal or pathogenic bacteria could promote tumor cell invasion and angiogenesis, thereby potentially influencing tumor metastasis.^129^ In addition, exposure of cryptic‐like intestinal epithelial cells to *E. coli* induced Hypoxia‐inducible Factor 1 alpha (HIF‐1α) protein expression, which further enhanced the expression of VEGF, a ligand that promotes angiogenesis.^130^

#### 3.2.5 Intratumor microbiome regulates stem cell niche

Importantly, the intratumor microbiome also actively contributes to regulating cancer stem cell niche. During early life after birth, gut microbiota colonization, especially *Lactobacillus*, is vital in regulating stem cell niche and orchestrate stem cell differentiation and tissue development via influencing intestinal macrophages.^131^ In CRC, CRC‐enriched microbiota metabolite isovalerate elevated 5‐HT production in intestinal nerve cells, which constituted the stem cell niche to enhance the stemness of cancer stem cells and promoted CRC progression.^132^ Similar effect was also found in physiological stem cell self‐renewal.^133^ *F. nucleatum*‐derived formate drove CRC tumor invasion by triggering AhR signaling and increasing cancer stemness.^134^

Despite the great research advances of intratumor microbiome in regulating the tumor microenvironment, we should be aware that most of researches focused on the immune microenvironemnt, and few researches uncovered the specific mechanisms. The tumor microenvironment is rather complex and is composed of many components. The microbiome may also interact with the other components, such as fibroblasts, etc., which worths further investigations.

## 4 INTERACTIONS BETWEEN INTRATUMOR MICROBIOME AND CANCER THERAPY

### 4.1 Cancer therapy modulates the composition of intratumor microbiome

**FIGURE 3:**
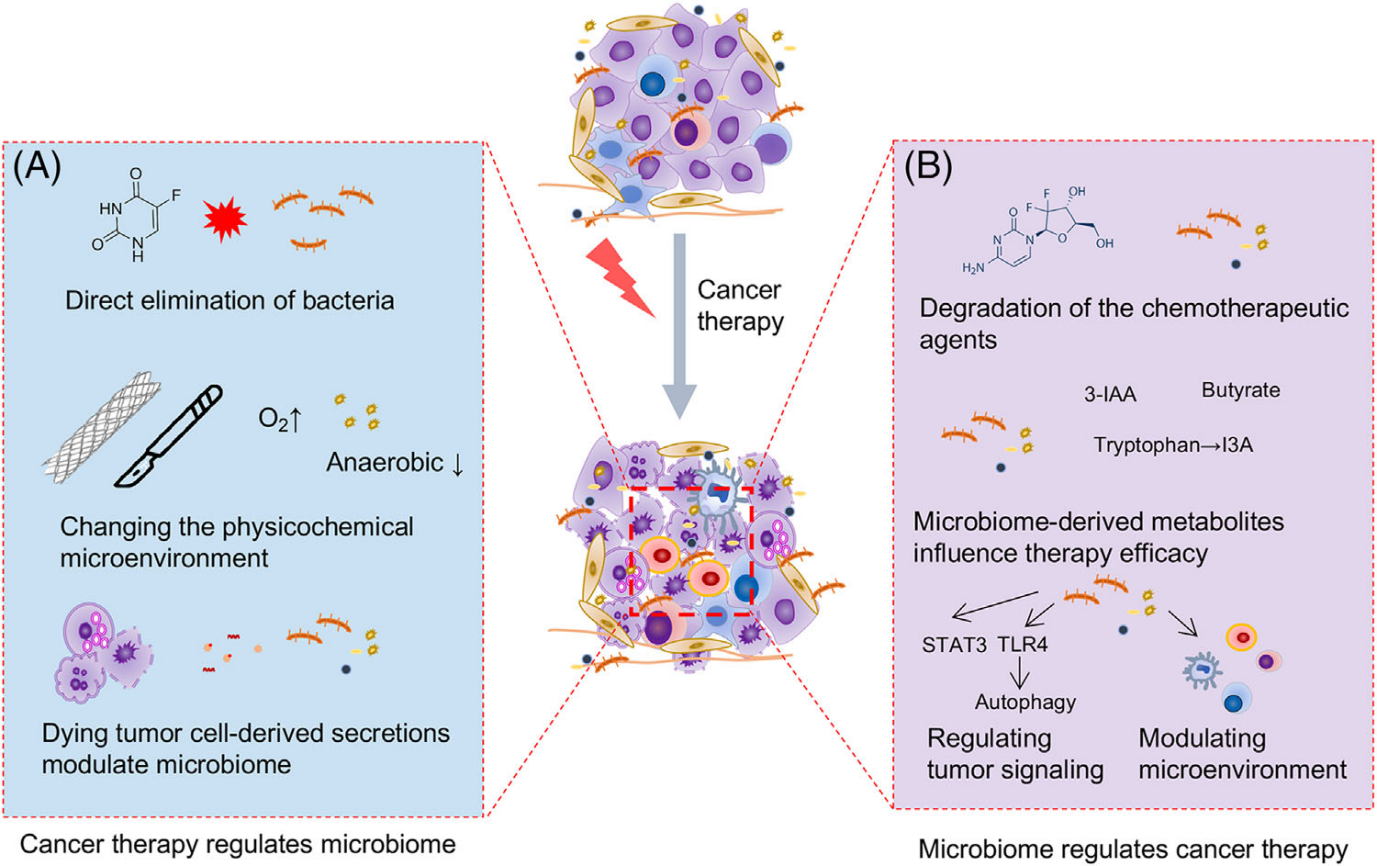
The interaction between intratumor microbiome and cancer therapy. (A) The adopted cancer therapy strategies may regulate the composition of intratumor microbiome, including directly eliminating bacteria by chemotherapy and changing the physicochemical microenvironment by surgery or other methods, and therapy‐induced dying tumor cells may regulate the microbiome via secreting signal molecules or cellular components. (B) The intratumor microbiome may further influence therapy efficacy by directly degrading the chemotherapeutic agents or secreting metabolites. Moreover, the role of intratumor microbiome in regulating tumor biology may further contribute to influencing the efficacy of cancer therapy.

While treating cancers, the adopted cancer therapeutic regimens could significantly change the microenvironment, including the intratumor microbiome (Figure 3A). After pancreaticoduodenectomy, the composition of microbiome in fecal samples, pancreatic fluid, bile, and jejunal contents was significantly changed, with enriched *Klebsiella* and *Bacteroides* and depleted anaerobic taxa (e.g., *Roseburia* and *Faecalibacterium*).^135^ These changes indicate that the intratumor microbiome could also be changed based on the former discussion that the resident microbiome is the important source of intratumor microbiome. Biliary stent placement and neoadjuvant chemotherapy with a combination of gemcitabine and paclitaxel were associated with significantly greater abundance of microbiota from the family *Enterobacteriaceae* in the tissue.^136^ Furthermore, the bacteria were found to exploit dying cell‐released nutrients to accelerate growth. Dying cells, mainly apoptotic cells induced by several apoptotic triggers, accelerated the growth of pathogenic *Salmonella* via secreting pyruvate, which was mediated by the pyruvate formate‐lyase‐encoding *pflB* gene.^50^ Upon cytotoxic therapy, it is inevitable to generate massive numbers of dying tumor cells, which is one of the main drivers of tumor repopulation and therapy resistance.^137^, ^138^, ^139^ It may be that the effect of dying tumor cells is partially mediated by the changed intratumor microbiome. Taken together, these results reveal that the cancer therapeutic strategies could change the composition of microbiome, which may partly be due to the change in the tumor microenvironment induced by the therapy. These changes may further influence the efficacy of therapy. However, related studies are relatively few, and further research is of high value.

### 4.2 Intratumor microbiome actively influences the efficacy of cancer therapy

Intratumor microbiome has recently been revealed to be associated with or to directly influence the therapy response by many studies. The metabolic ability and the metabolites of microbiome are vital in regulating therapeutic effects. Moreover, the regulation of microbiome on tumor cell biology also contributes to tumor therapy response (Figure 3B).

The effects of many chemotherapeutic drugs including gemcitabine, fludarabine, cladribine, etc. were revealed to be attenuated or enhanced by the bacteria that were commonly found in tumor tissues. These effects were at least partly mediated by the modification of chemical structure of drugs by bacteria.^140^ Further researches revealed that intratumor *Gammaproteobacteria* conferred gemcitabine resistance to cancers, including colon cancer, pancreatic cancer, etc., by expressing the bacterial enzyme cytidine deaminase (CDD_L_). And most of the pancreatic cancer tissues have been detected with bacteria, the majority of which are *Gammaproteobacteria*,^141^ indicating the underlying mechanisms of poor response to chemotherapy in pancreatic cancer. On the other hand, microbiota‐derived tryptophan metabolite indole‐3‐acetic acid enhanced the chemotherapeutic effects of pancreatic cancer via elevating the accumulation of Reactive Oxygen Species (ROS) and downregulating autophagy in cancer cells.^142^ And intratumor *Clostridium butyricum* and its metabolite butyrate could promote susceptibility to ferroptosis in pancreatic cancer,^143^ which is a promising way to enhance therapeutic effects.^144^ In breast cancer, probiotic *Lactobacillus reuteri* could translocate to intratumor tissues, which catalyzed the dietary tryptophan into I3A and promoted interferon‐γ (IFN‐γ)‐producing CD8 T cells, thereby bolstering the effect of immune checkpoint inhibitors.^145^ These results highlight the important role of bacteria‐derived metabolite in regulating therapy responses.

Moreover, *F. nucleatum* in CRC activated TLR4 to enhance autophagy in cancer cells and thus conferring chemoresistance.^146^ Intratumor lipopolysaccharide (LPS)‐activated NF‐κB–IL6–STAT3 axis facilitated prostate cancer proliferation and docetaxel chemoresistance, which was caused by antibiotic use that elevated the abundance of *Proteobacteria* and gut permeability.^147^ Furthermore, the influence of microbiome in regulating the efficacy of immunotherapy was highly appreciated.^148^ Certain microbes showed the potential to predict the response to immune checkpoint blockade therapy with an AUC of 89%.^107^ Although most of the research concerning immunotherapy was conducted in gut microbiome, the potential effects of intratumor microbiome should be noted due to the aforementioned fact that the intratumor microbiome actively regulates the immune landscape.

## 5 EXPLOITING THE INTRATUMOR MICROBIOME AS THERAPEUTIC TARGETS

Since the intratumor microbiome plays vital roles in regulating tumor cell biology and the tumor microenvironment as well as the therapy response, selectively modulating the tumor microenvironment has become a research interest. Moreover, the tumor microenvironment is hospitable for the residing of certain bacteria; thus, engineering the bacteria to exploit them as therapeutic weapons has also shown promising potentials. However, due to the great diversity existing among different kinds of tumors, it is almost impossible to target the intratumor microbiome using an identical method. Thus, there are numerous attempts to manipulating the intratumor microbiome.

### 5.1 Targeting the intratumor microbiome by antibiotics

As most of the microbiome within tumors are pro‐tumoral, the first attempt to control the influence of microbiome is applying antibiotics, which have shown some beneficial effects. Treatment with antibiotic metronidazole reduced *Fusobacterium* load in PDX tumor‐bearing mice, and the proliferation of cancer cells and overall tumor growth were also inhibited after using antibiotics.^4^ Prolonged antibiotic treatment‐induced microbiota diversity reduction was associated with higher intratumor immune response and a better antitumor effect induced by neoantigen cancer vaccines.^149^ And many experimental investigations have revealed the beneficial effects of antibiotic use.^3^, ^46^, ^85^, ^98^

However, in a prospective multicenter cohort study, prior antibiotic therapy impaired the efficacy of immune checkpoint inhibitor therapy (Hazard Ratio (HR), 7.4; 95% confidence interval [CI], 4.3–12.8; *p* < 0.001) and shortened the survival time of patients (HR, 7.4; 95% CI, 4.2–12.9).^150^ Prior to the immune checkpoint inhibitor era, antimicrobial prescription was associated with inferior overall and cancer‐specific survival in breast cancer patients.^151^ And systematical use of antibiotics in gemcitabine chemotherapy of pancreatic cancer patients led to more severe side effects, including increased risk of anemia, thrombocytopenia, leukopenia, neutropenia, and gastrointestinal adverse events.^152^ These facts highlight the need for caution during intervention of bacteria using antibiotics. Moreover, antibiotic use could otherwise alter the microbiota composition, resulting in dysbiosis and increased risk of further infection and may promote the spread of drug‐resistant pathogens.^153^, ^154^, ^155^ And the disruption of gut microbiome by antibiotics may otherwise lead to tumor progression or therapy resistance. Disruption of gut microbiota using antibiotics enhanced epithelial ovarian cancer progression and cisplatin resistance; metabolomics analyses revealed that this was partly due to the disruption of gut microbiota‐derived metabolites.^156^ Disruption of the gut microbiota impaired the response of subcutaneous tumors to CpG‐oligonucleotide immunotherapy and platinum chemotherapy via modulating myeloid‐derived cell functions in the tumor microenvironment.^157^ Of note, the condensed tumor microenvironment may not be suitable for antibiotics to penetrate well, such as chemotherapeutic agents. Thus, there may be gaps between experimental and clinical conditions. Hence, attempting either to selectively target specific pathogens without perturbing the physiological microbiome or to re‐establish commensal communities is a promising direction.^158^

### 5.2 Strategies of specific modulating the intratumor microbiome

The capacity of bacteriophages to kill specific bacteria and modulate immunity makes them suitable candidates to manipulate the tumor microbiome and enhance the efficacy of tumor control.^159^ However, due to differences in anatomic and the tumor microenvironment, the administration and consequences of bacteriophages may be different, but the related studies are still lacking. Supplementation with probiotics is a feasible way to modulate the intratumor microbiome, especially in CRC. A consortium of 11 bacterial strains from healthy human donor feces was capable of robustly inducing IFN‐γ‐producing CD8^+^ T cells in the intestine.^160^ Oral application of a mix of four *Clostridiales* strains, or single application of *Roseburia intestinalis* or *Anaerostipes caccae*, prevented and even successfully treated CRC as stand‐alone therapy via CD8^+^ T‐cell‐dependent manner in mice.^161^ In addition, using prebiotics to potentiate the diversity of microbiome could also be a way to modulate the microbiome. A bilberry anthocyanin combo containing chitosan and low molecular citrus pectin enriched the subdominant species, increased both the concentration and the proportion of butyrate in feces and enhanced intratumor CD8^+^ T‐cell infiltration, which led to the best control of tumor growth.^162^ Prebiotic changed the gut microbiota and thus enhanced the antitumor immunity.^163^ In addition, administration of postbiotics may have superiority in terms of safety relative to their parental live cells.^164^ Interestingly, high‐salt diet enhanced the intratumor colonization of *Bifidobacterium* by elevating gut permeability, which promoted NK‐cell‐mediated antitumor immunity.^165^ However, we should be aware that these experiments were mainly performed on CRC, due to easy manipulation through the oral route. Other methods should be considered in other cancer types. Although these strategies mainly target the gut microbiome, they may indirectly influence or provide some clues to modulate the intratumor microbiome. Of note, systematical adiministration of *Bifidobacterium* bacteria would accumulate in the tumor tissues and stimulate STING signaling, which further increased the cross‐priming of dendritic cells after anti‐CD47 treatment.^166^ And oral consumption of *Lactobacillus* decreased tumor growth of triple‐negative breast cancer in a syngeneic breast cancer model.^167^ These results highlight the feasibility of modulating intratumor microbiome via oral route.

### 5.3 Exploiting intratumor microbiome as a weapon to fight against cancer

In addition, taking advantage of the characteristics of intratumor microbiome to suppress cancer is also under investigation. Tumor‐resident microbiota showed great tumor‐homing potential; thus, they could be potentially tumor‐targeting weapons. Some bacteria isolated from the tumors showed preferential growth and proliferation within a targeted tumor milieu, and effectively caused immune cells to infiltrate the tumor and provoked strong anti‐cancer responses in various syngeneic mouse models.^168^ Similar effect was also used to generate bacteria with intratumoral release of nanobodies targeting PD‐L1 and CTLA‐4, and administration of the bacteria showed enhanced therapeutic response.^169^ *Bacillus toyonensis* is normally presented in healthy people but absent in the feces of CRC patients. *B. toyonensis*‐derived hemolysin BL has strong effects on killing tumor cells by causing cell membrane disruption, blebbing, and leakage of cytoplasmic contents, which has promising effects in controlling CRC.^170^ *Neospora caninum* inhibited B16F10 melanoma by activating potent immune responses and directly destroying cancer cells.^171^ Moreover, direct intratumor injection of bacteria has presented some enlightening facts, and is also a feasible way. Intratumor injection of attenuated strain of *Clostridium novyi* showed promising effects in suppressing brain tumor.^172^ Further clinical study showed the therapeutic potential of intratumor injection in treatment‐refractory advanced solid tumors.^173^ However, the high risk of adverse effects should be noted.^173^ Interestingly, as the recurrent bacterial peptides in tumors being identified,^106^ tumor‐specific CD8^+^ T cells with a second T‐cell receptor (TCR) that recognizes a bacterial antigen were generated, and treatment of solid tumors with the dual‐specific T cells and intratumor injection of bacteria showed therapeutic potentials.^174^ Moreover, clinical study showed that intratumor injection of engineered bacteria could regulate the tumor signaling and tumor microenvironment.^175^ And there are opportunities that may promote the abscopal effects.^176^

### 5.4 Ongoing findings of ways to regulate intratumor microbiome

Moreover, modulating the intratumor microbiome via other mechanisms has been investigated. Administration of adhesive hydrogel incorporating silver nanoparticles (which inhibited the growth of bacteria competing with *Peptostreptococcus*) alongside the intratumor delivery of the bacterium *P. anaerobius* synergized with PD‐1 inhibition greatly enhanced the efficacy of PD‐1 inhibition.^177^ Interestingly, screening of small‐molecule inhibitors revealed that many antineoplastic agents, including 5‐FU, were the potential inhibitors of *F. nucleatum*, clearing of which should enhance the efficacy of CRC treatment. However, the 5‐Fluorouracil (5‐FU) was metabolized by intratumor bacteria, including *E. coli*, to relieve the effect of 5‐FU,^178^ indicating that the intratumor microbiome may have synergistic effects. In addition, modulation of microbiome by traditional medicine, such as tranditional Chinese medicine, has been noted^179^, ^180^; although the investigation concerning intratumor microbiome is still lacking, it is an interesting research area that worths more efforts.

## 6 DISCUSSION

Recently, numerous studies have revealed that bacteria could reside in the tumor microenvironment, even tumors that are not directly in contact with the external microenvironment. These intratumor microbiome have been validated to be associated with the prognosis of patients, and participated in modulating tumor initiation, progression and therapy response. In this review, we highlighted that the intratumor microbiome, like the other components in the tumor microenvironment, is actively shaped by the tumor cells and surrounding microenvironment and conversely contributes to regulating the tumor biology. Specifically, the characteristics of the tumor microenvironment, including hypoxia, immune suppression, etc., are specifically suitable for the colonization of microbiome, and the interaction between tumor microenvironment and bacteria, such as molecular binding or secretions from the tumor cells enhances the colonization of microbiome. Conversely, the microbiome promotes cancer development via modulating genetic mutational landscape, the phenotype of genes, cellular signaling, cell metabolism, etc., and reprogramming the tumor inflammatory, immune, and angiogenic microenvironment as well as cancer stem cell niche (Table 2). Of note, therapeutic strategies could change the composition of intratumor microbiome, which reversely influences the efficacy of these therapies. Based on these findings, targeting the intratumor microbiome or engineering the microbiome to fight against cancer has become a feasible way. Specifically, targeting the harmful bacteria or supplement of probiotics showed therapeutic potential. However, challenges exist in the lack of powerful strategies to selectively eradicate pathogens, and accompanied side effects should be noted. Taking advantage of the tumor‐homing features of the intratumor microbiome, engineering them to specifically kill cancer cells or release effective molecules provide potential ways to control tumors, but there are relatively few related investigations. More targeted therapeutic strategies based on deciphering the genetic and molecular basis of tumor cell–microbiome interaction should be the future investigational direction. These results strongly support that the intratumor microbiome is the vital component of the tumor microenvironment and promotes cancer development.

**TABLE 2:** Mechanisms of intratumor microbiome in influencing the tumor biology.

Mechanism	Tumor type	Microorganism	Mediator and specific effect	References
Secretion of metabolites	Colorectal cancer	*Escherichia coli*	Colibactin; inducing DNA damage, mutation, and tumor initiation	^65^, ^67^
	Colorectal cancer	*E. coli*	VirF; disrupting the gut vascular barrier	^104^
	Colorectal cancer	*Bacteroides fragilis*	*B. fragilis* toxin; inducing DNA damage and mutation	^65^
	Colorectal cancer	Microbiome	Gallic acid; reinstate the TCF4–chromatin interaction and the hyperactivation of Wnt signaling	^80^
	Colorectal cancer	Microbiome	Isovalerate; elevating 5‐HT production in intestinal nerve cells to constitute the stem cell niche	^132^
	Colorectal cancer	Gut microbiota	Butyrate; functioning as a HDAC inhibitor to stimulate histone acetylation and affect apoptosis and cell proliferation	^90^
	Pancreatic cancer	Microbiome	3‐IAA; elevating accumulation of ROS and downregulating autophagy in cancer cells to promote therapy effect	^142^
	Pancreatic cancer	*Clostridium butyricum*	Butyrate; promoting ferroptosis susceptibility	^143^
Metabolizing compounds	Oral cancer	*Neisseria subflava*, *Streptococcus mitis*, *Candida albicans*, *Glabrata*, etc.	Converting alcohol into carcinogen acetaldehyde	^70^, ^71^
	Pancreatic cancer, colon cancer	*Gammaproteobacteria*	Bacterial enzyme cytidine deaminase; metabolize gemcitabine to confer chemoresistance	^141^
	Breast cancer	*Lactobacillus reuteri*	Catalyzing the dietary tryptophan into I3A and promoting interferon‐γ‐producing CD8 T cells	^145^
Molecule binding with cancer cells	Colorectal cancer	*Fusobacterium nucleatum*	Bind to E‐cadherin, activates β‐catenin signaling	^80^
	Colorectal cancer	*Peptostreptococcus anaerobius*	Surface protein PCWBR2 directly bind to α_2_/β_1_ integrin and activate the PI3K–Akt pathway	^81^
	Multiple cancers	*F. nucleatum*	Fap2 binds to human TIGIT and inhibit immune activity of NK and T cells	^114^
Inducing signaling changes in cancer cell	Breast cancer	*Staphylococcus*, *Lactobacillus*, and *Streptococcus*	Reorganizing actin cytoskeleton to enhance resistance to fluid shear stress	^85^
	Oral cancer	*F. nucleatum*	Activating intracellular autophagy pathways and promoting migration and metastasis.	^86^
	Colorectal cancer	*F. nucleatum*	Activating STING signaling and promoting PD‐L1 expression	^110^
	Colorectal cancer	*F. nucleatum*	Activating TLR4 to enhance autophagy in cancer cells and conferring chemoresistance	^146^
Modulating the tumor microenvironment	Melanoma	Intratumor microbiome	Bacteria specific peptides are presented by the tumor cells in both HLA‐I and HLA‐II	^106^
	Lung cancer	Commensal microbiota	Modulating the immune microenvironment to promote carcinogenesis	^3^
	Colorectal cancer	*F. nucleatum*	Recruiting tumor‐infiltrating myeloid cells	^43^
	Colorectal cancer	*F. nucleatum*	Promoting CCL20 expression in tumor cell and inducing M2 macrophage polarization	^121^
	Colorectal cancer	*Akkermansia muciniphila*	Inducing the enrichment of M1‐like macrophages in an NLRP3‐dependent manner	^123^
	Colorectal cancer	*Porphyromonas gingivalis*	Recruiting myeloid cells and creating a proinflammatory tumor microenvironment.	^113^
	Pancreatic cancer	*P. gingivalis*	Recruiting neutrophils and creating a proinflammatory microenvironment	^115^
	Gastric carcinoma	*Helicobacter pylori*	Increasing VEGF expression and neo‐angiogenesis	^128^
DNA transfer	Human endothelial cell	*Bartonella henselae*	Conjugative DNA; inducing genetic mutation	^75^

Abbreviations: 3‐IAA, indole‐3‐acetic acid; HDAC, histone deacetylase.

Of note, as the important component of our body, the diversity of bacterial genes is 100 times greater than that of our genes, which contain numerous genes that do not exist in human cells and may extend the ability of human body.^181^, ^182^ The metabolic capacity that the human cells do not possess and their metabolites may play vital roles in pathophysiological processes. For example, N‐acyl amide synthase genes were enriched in gastrointestinal bacteria and the lipids that they encode interacted with G‐protein‐coupled Receptors (GPCRs) to regulate gastrointestinal tract physiology.^183^ Under the circumstance of cancer therapy, the *Gammaproteobacteria* bacterial enzyme CDD_L_ in pancreatic cancer inactivated the chemotherapy agent gemcitabine.^141^ Moreover, 1952 uncultured candidate bacterial species were identified by metagenome sequencing, which substantially expanded the known species repertoire of the collective human gut microbiota, and these bacteria encoded hundreds of newly identified biosynthetic gene clusters and possessed a distinctive functional capacity.^184^ These results strongly support the great potential of microbiome in regulating tumor biology, but their capacity and the underlying molecular mechanisms are not fully recognized. Further research concerning the genetic and molecular basis of the microbiome is of high value.

In addition, besides bacteria reside directly in the tumor microenvironment, microbiota reside in gut also regulate the tumor microenvironment by secreting signal molecules directly or indirectly. Gut microbiota‐derived STING agonists induced IFN‐I production by intratumor monocytes to regulate macrophage polarization and NK cell‐dendritic cell crosstalk and thus enhancing antitumor immunity.^185^ While gut microbiome‐derived butyrate was found to inhibit STING signaling activation following radiotherapy, which abrogated radiotherapy‐induced tumor‐specific cytotoxic T‐cell immune responses without directly protecting tumor cells from radiation,^186^ highlighting the context‐dependent role of different composition of microbiome. Commensal microbe‐derived butyrate was revealed to induce the differentiation of colonic regulatory T cells, possibly via enhancing histone H3 acetylation in the promoter and conserving non‐coding sequence regions of the Foxp3 locus.^187^ Gut microbiome‐mediated primary‐to‐secondary bile acid conversion controlled the liver‐selective antitumor effect via regulating CXCL16 expression in liver sinusoidal endothelial cells.^188^ These results also strongly support the remote regulatory role of the microbiome, even though they do not directly interact with tumor tissues. This reminds us that we should treat the tumor as a systematical disease and broaden our view when managing cancers.

Although recent researches have mainly focused on the bacteriome, the role of other microorganisms should also be noted. The recent few years have also witnessed that besides bacteria, other microorganisms are also presented in the tumor microenvironment and showed great potential in modulating cancer progression. The fungal DNA and cells were presented across many major human cancers, although at low abundances.^189^, ^190^, ^191^ In both pancreatic cancer patients and mouse models, the fungi were found to be enriched by about 3000‐fold compared with the normal pancreas, with *Malassezia* spp. as the most abundant species. *Malassezia* accelerated tumor oncogenesis and progression in a mannose‐binding lectin‐dependent manner.^192^ Moreover, intratumor fungi were one of the driving forces of IL‐33 secretion in pancreatic cancer, which recruited Th2 cells and innate lymphoid cells 2 to promote cancer progression.^193^ Of note, gut commensal fungi and bacteria might have opposite influences on antitumor immunity following radiation, with bacteria promoting the response and fungi reducing it.^194^ In soft tissue sarcoma, a strong positive correlation between viral relative abundance and NK cell infiltration was observed, and more NK infiltration was associated with superior metastasis‐free and OS,^195^ highlighting the potential roles of intratumor virus in tumor biology. And virus may bring another level of messages in the tumor microenvironment.^196^ These results remind us that other microorganisms also possess great potential in modulating tumor biology, but we should be aware that reliable investigational methods and results to illustrate the cause–effect relationship between tumor cells and these microorganisms are still lacking. This is an ongoing area that worths more efforts.

**FIGURE 4:**
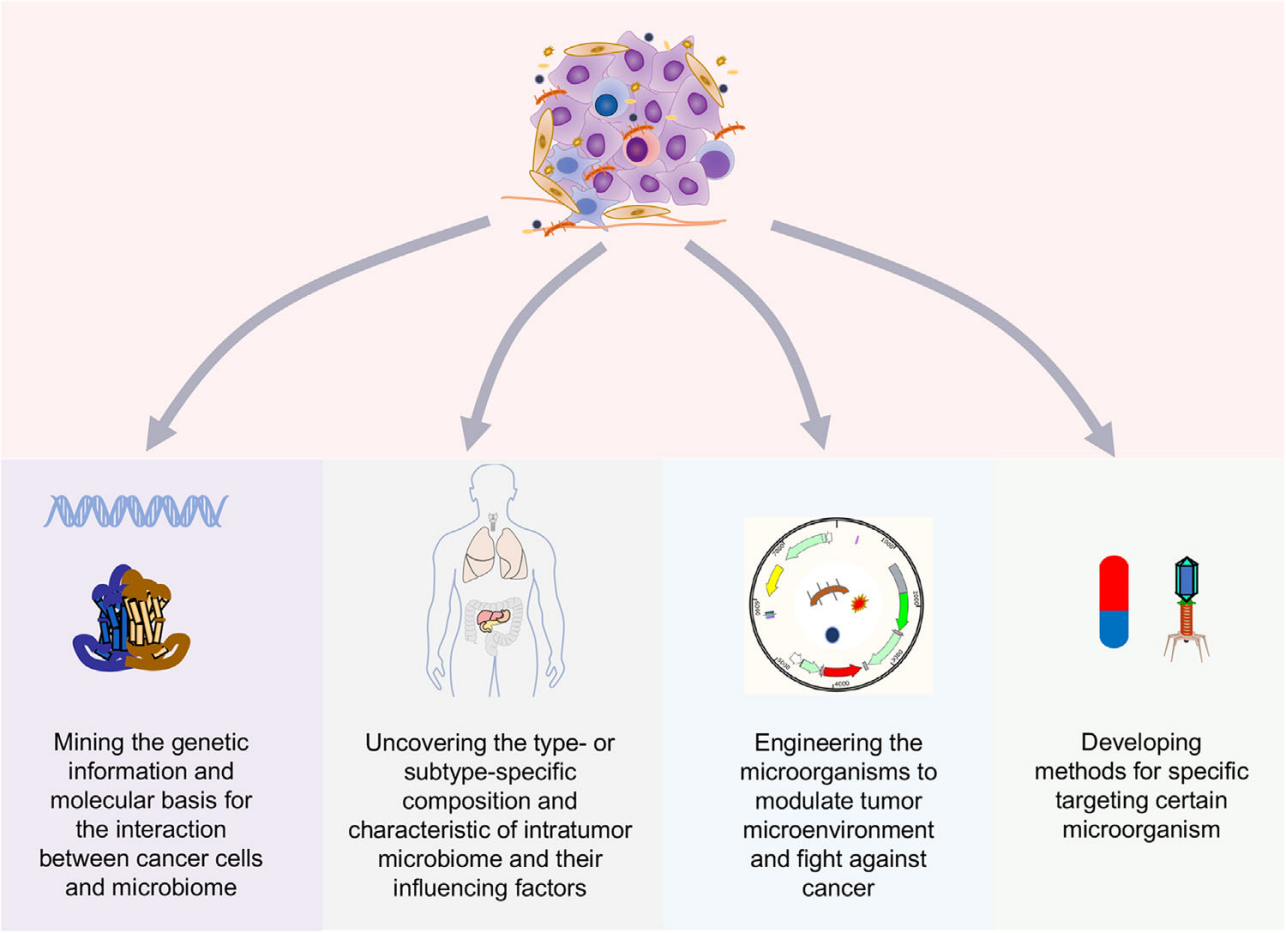
Potential future investigational directions concerning intratumor microbiome. The microbiome contains large amount of unrecognized genetic information and the molecular basis for the interaction between microbiome and the tumor cells has just begun to be uncovered. Thus, mining the underlying mechanisms is of great value. Moreover, the tumor microenvironment is significantly different between different tumor types or even subtypes; this may account for the varied composition and function of intratumor microbiome. Thus, deciphering the context‐dependent messages is of great value. Furthermore, exploiting the tumor microbiome also shows promising effects in controlling cancer; engineering the microorganisms is a potential feasible way. Last, developing reliable methods to specifically upregulate or deplete certain microorganisms, such as *Fusobacterium nucleatum*, is also a direction worth trying.

Nevertheless, there are several problems that restrain the development of the research area. First, the contamination of external bacteria is quite a problem during the long process from specimen collection to sequencing.^197^, ^198^ Stringent sterile procedure and suitable control should be adopted to get the most reliable results.^2^ New detection methods to test the accuracy are a way to validate the results. For example, by adopting comprehensive histological imaging, it was found that bacteria in the glioma were mostly localized near nuclear membranes or in the intercellular space.^199^ In addition, the consistency of formalin‐fixed paraffin‐embedded (FFPE) tissues and fresh frozen tissues in retrieving the composition of core bacteria within breast tumors highlights the meaning and reliability of retrospective studies,^200^ which could simplify the intratumor studies and accelerate the research progress by resuscitating massive messages embedded in FFPE. Moreover, most of the results just uncover the correlation between microbiome and cancers, but the cause‐effect evidences are still lacking. Thus, we could not know whether the changes in the microbiome in the tumor microenvironment are the passengers during cancer development or actively participate in the process in many researches. Furthermore, the intratumor microbiome is also influenced by many factors. Race is an important factor influencing the composition of intratumor microbiome, which is also significantly associated with the different tumor biology.^201^, ^202^ And diet could be an influencing factor in changing the composition of intratumor microbiome.^203^ Mediterranean diet consumption led to increased mammary gland *Lactobacillus* abundance compared with western diet‐fed monkeys^204^; while *Lactobacillus* was revealed to be downregulated in breast cancer tissues and acted as a potential antitumor microbe.^205^ A high‐salt diet was revealed to enhance the intratumor colonization of *Bifidobacterium* by elevating the gut permeability.^165^ These phenomena indicate that intratumor microbiome is regulated by many factors, and more stringent control conditions should be adopted when performing studies; and the microbiome is amendable for management, and strategies other than killing the bacteria could be considered (Figure 4).

**FIGURE 5:**
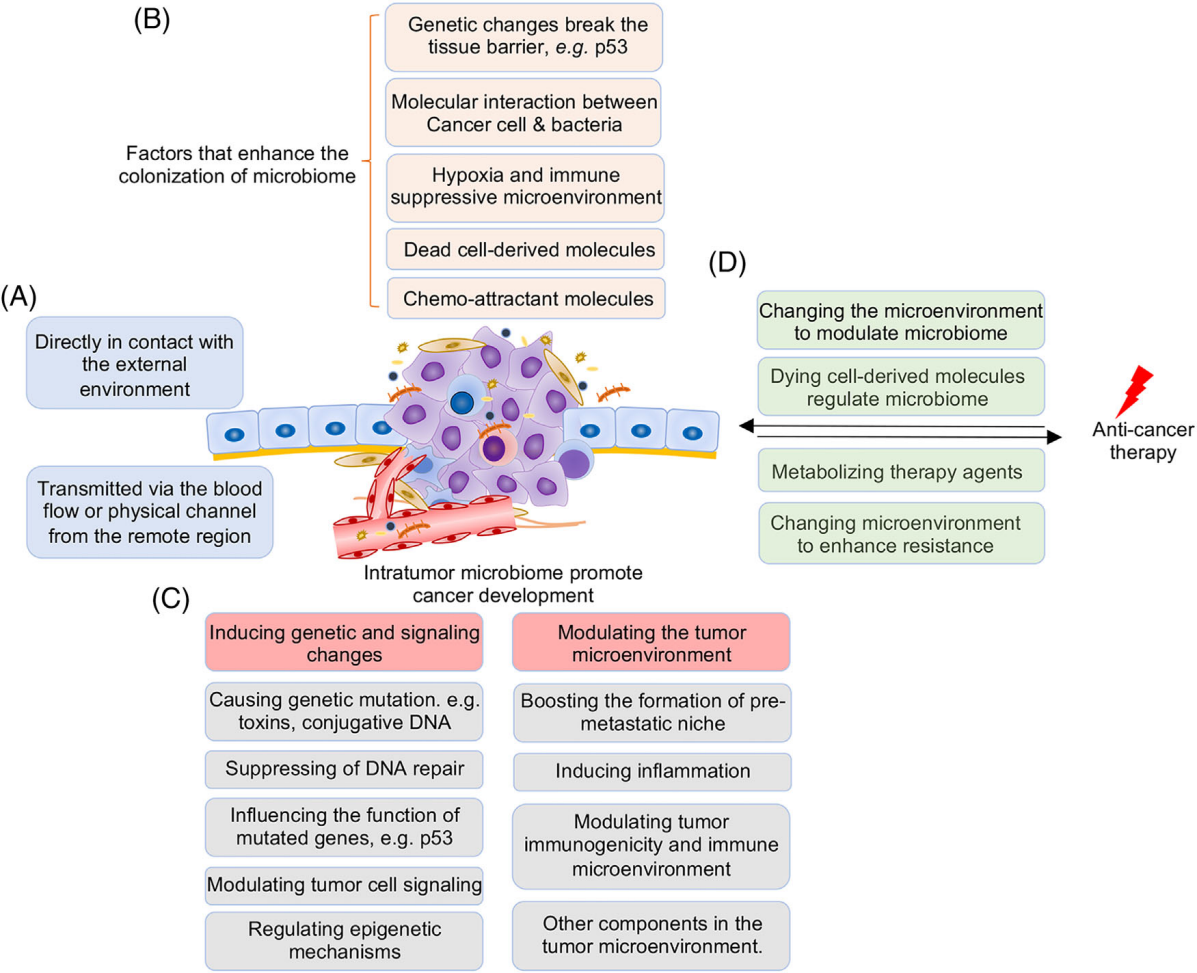
Interplay between intratumor microbiome and the tumor microenvironment. (A) Routes that microbiome enters the tumor microenvironment. Tumors that directly connect with the external microenvironment could be colonized by microbiome from the resident bacteria. Microbiome could also transmit from the blood stream or other physical channels. (B) The tumor microenvironment is hospitable for the colonization of microbiome. The characteristics of the tumor microenvironment select suitable bacteria to reside and the interaction of microbiome with the tumor microenvironment promotes microbiome colonization. (C) Intratumor microbiome promotes cancer initiation and progression. The intratumor microbiome promotes cancer development via inducing genetic and signaling changes and modulating the tumor microenvironment through various mechanisms as presented. (D) Mutual regulation of the intratumor microbiome and the adopted therapy. The cancer therapy strategies change the composition of intratumor microbiome, while microbiome also actively influences the efficacy of cancer therapy.

In summary, we demonstrated that intratumor microbiome is a vital component of the tumor microenvironment and interacts with tumor cells and the surrounding microenvironment, which influences tumor development and therapy response and is a therapeutic target (Figure 5). More stringent experimental conditions, developing context‐dependent strategies to uncover the genetic, and molecular basis for better understanding of the intratumor microbiome is the basis for further investigations.

## AUTHOR CONTRIBUTIONS

M.J. conceived and drafted the manuscript, drew the figures, and discussed the concepts of the manuscript. Z.Y. and J.D. conceived and drafted the manuscript, and discussed the concepts of the manuscript. A.Y. and W.C. supervised the process, provided valuable discussion and revised the manuscript. T.W., Z.J., Y.Y., and K.N. provided valuable discussion and revised the manuscript. All authors have read and approved the final manuscript.

## CONFLICT OF INTEREST STATEMENT

The authors declare they have no conflicts of interest.

## ETHICS STATEMENT

Not applicable.

## Data Availability

Not applicable.
